# Supplementary material for: The Usability of Neurological Occupational Therapy Case Studies Generated by ChatGPT
Source: Healthcare (Basel). 2025 Jun 4;13(11):1341. doi: 10.3390/healthcare13111341 (PMC12155196; doi:10.3390/healthcare13111341)
Supplement: Supplementary file 1 [file healthcare-13-01341-s001.zip › healthcare-3645885-supplementary.pdf]

Supplementary Table S1.

Table S1. The finalized prompt for ChatGPT-generated cases.

Variable	Content
Prompt	From now on, we will develop clinical cases for occupational therapy students. The patient group is limited to patients with central nervous system damage such as stroke or spinal cord injury. Your case must include three items: The first item is a scenario, and the scenario includes the patient's gender and age, the patient's time (considering the patient's future, whether to go to another hospital or be discharged and go home, etc.), and the environment (whether there are stairs at home, etc.)), role (e.g., what are the important roles), adaptive (e.g., is it acceptable to use a cane for walking), motivational (e.g., are values, interests, and goals motivating one to do a task?), and social (e.g., other What kind of relationships do you have with people, etc.), social institutional (financial environment and social support, etc.), ability (what are your physical, cognitive, psychological impairments, etc.), cultural (where, how, and what you do?) etc.) and task-related aspects (what tasks are desired, what tasks are difficult, etc.) should be included and should be presented in an integrated manner rather than separated. The second item is occupational therapy evaluation results, which should include the patient's subjective information and objective occupational therapy evaluation results. The third item is a clinical question. Based on the previous two items, it should include 10 questions related to establishing occupational therapy goals and plans and points to be considered when conducting occupational therapy.
